# Effect of denosumab versus zoledronic acid on calcium levels in cancer patients with bone metastasis: A retrospective cohort study

**DOI:** 10.1177/1078155218820927

**Published:** 2019-01-07

**Authors:** Sahar M Nasser, Arwa Sahal, Anas Hamad, Shereen Elazzazy

**Affiliations:** Pharmacy Department, National Center of Cancer Care & Research, Hamad Medical Corporation, Doha, Qatar

**Keywords:** Denosumab, zoledronic acid, bone metastasis, cancer, hypocalcemia

## Abstract

**Objective:**

To identify the incidence of hypercalcemia and hypocalcemia in zoledronic acid and denosumab groups. Secondary objective was to determine the correlation between calcium supplement and calcium level control.

**Methods:**

An observational retrospective cohort study was conducted by reviewing patient electronic records, laboratory results, and medication charts from 1 August 2015 to 31 July 2016. Adult cancer patients who were diagnosed with bone metastasis secondary to a solid tumor or multiple myeloma and who received either zoledronic acid or denosumab were included. Other indications for bone targeting agents were excluded. Data of bone targeting agents administration encounters were collected, evaluated, and analyzed.

**Results:**

A total of 1141 encounters (for 271 patients) were included. The incidence of hypocalcemia was higher in denosumab compared to zoledronic acid group (5.5% vs. 3.1%, OR = 0.55, 95% CI [0.3–1.0]; P = 0.05). Hypercalcemia incidence was also higher in denosumab group (8.5% vs. 3.1%, OR = 2.9, 95% CI [1.68–5.03]; P < 0.0001). Breast cancer was the most common malignancy associated with hypocalcemia (27.3%) followed by ovarian cancer (25%) and multiple myeloma (22.7%). The risk of developing hypocalcemia was reduced by 16% in patients receiving calcium supplementation (RR = 0.84, 95% CI [0.55–1.20]; P = 0.39).

**Conclusion:**

Denosumab use was associated with higher rates of both hypercalcemia and hypocalcemia compared to zoledronic acid. Adequate supplementation with calcium substantially reduced the risk of hypocalcemia. Our results highlight the importance of taking preventative measures upon bone targeting agents initiation and during treatment including regular monitoring of calcium levels and providing supplements accordingly.

## Introduction

Cancer and chemotherapy can compromise bone health by the interaction between tumor and bone cells. This cause disruption of normal bone metabolism by increasing osteoclast activity leading to bone resorption.^1^ Patients with metastatic bone disease or multiple myeloma commonly experience osteoclast-mediated bone destruction and its associated complications. These complications are known as skeletal-related events (SREs).^2^ These events are defined by five major complications of tumor bone disease: pathological fractures, need for radiotherapy to the bone, need for bone surgery, spinal cord compression, and hypercalcemia.^3,4^

Bone-targeting agents (BTA) like zoledronic acid (ZA) and denosumab (DE) are approved for the prevention of SREs in patients with bone metastases (BM) including hypercalcemia of malignancy (HCM).^5,6^ Intravenous bisphosphonates, such as ZA, have a direct apoptotic effect on osteoclasts and act as potent inhibitors of bone resorption and skeletal calcium release.^7^ ZA adverse effects include bone pain, flue-like symptoms, osteonecrosis of the jaw, dose-dependent nephrotoxicity, and hypocalcemia.^8^

The incidence of hypocalcemia associated with ZA is minimal, as shown by different studies.^9–11^ However, up to 40% of hypocalcemia cases were reported in individual patients with risk factors including renal failure, vitamin D deficiency, or pre-existing hypoparathyroidism.^12,13^ On the other hand, DE is a fully human monoclonal antibody with high affinity to human receptor activator of nuclear factor kappa-B ligand (RANKL).^6^ Through inhibiting RANKL, DE prevents bone destruction and reduces complications of BM in patients with advanced cancer.^14^ A recent study reported that hypocalcemia incidence was more frequent with DE than ZA, which is mostly due to the potency of RANKL inhibitors at reducing bone turnover, thus lessening the release of calcium into circulation.^15,16^

In the National Center for Cancer Care & Research (NCCCR), the only tertiary cancer care institute for adults in the State of Qatar, both ZA and DE are extensively used in the management of BM. Hypocalcemia has been observed with both ZA and DE. International guidelines do not favor one BTA over the other.^3,17^ Due to the differences in patients’ characteristics and treatment-related factors, the aim of this study was to assess the efficacy and safety of ZA and DE in relation to calcium level. The primary objective of this study was to evaluate the incidence of hypercalcemia (>2.55 mmol/l, > 10.2 mg/dl) and hypocalcemia (<2.1 mmol/l, < 8.4 mg/dl) among patients receiving ZA and DE. The secondary objectives included identifying other factors that contribute to hyper- and hypocalcemia, evaluating the grade of hypocalcemia and identifying the effect of calcium/vitamin D supplementation on calcium levels.

## Methods

This was a retrospective cohort study where patients’ electronic medical records, laboratory results, and medication charts were reviewed for the period of one year (1 August 2015–31 July 2016).

All adult cancer patients diagnosed with BM secondary to a solid tumor or multiple myeloma and who were receiving either ZA (standard dose of 4 mg IV every four weeks or adjusted based on renal function) or DE (standard dose of 120 mg SC every four weeks) were included in the study. Patients who were taking BTA for any other indication such as osteoporosis or HCM were excluded. Collected data included: age, gender, diagnosis, corrected calcium level, creatinine clearance, and calcium/vitamin D supplementation. Calcium and vitamin D supplements were collected from patient’s dispensing electronic records. These records reflect any dispensing occurred at any Hamad General Governmental Hospitals or health centers (main source of vitamin D and calcium supplement). Other sources like over-the counter or private and retail pharmacies will be accounted through documentation of patient reconciliation. Study was approved by the Hospital Research Committee at NCCCR and the Medical Research Centre at Hamad Medical Corporation. Our primary objective is to identify the incidence of hypercalcemia and hypocalcemia in ZA and DE groups. Secondary objective is to determine the correlation between calcium supplement and calcium level control.

### Study assessment

Corrected serum calcium level (adjusted for albumin levels) was collected before each administration encounter (four weeks between each encounter) and was analyzed to identify hypocalcemia or hypercalcemia. Hypocalcemia was defined as corrected calcium levels of < 2.1 mmol/l (8.8 mg/dl), and hypercalcemia was defined as corrected calcium levels of > 2.55 mmol/l (10.2 mg/dl). Hypocalcemia was divided into grades: Grade 1: corrected calcium level < 2 mmol/l (<8 mg/dl); Grade 2: corrected calcium level < 2–1.75 mmol/l (<8–7 mg/dl); Grade 3: corrected calcium level < 1.75–1.5 mmol/l (<7–6 mg/dl); and Grade 4: corrected calcium level < 1.5 mmol/l (<6 mg/dl). The incidence of hypocalcemia and its grade were defined according to the Common Terminology Criteria for Adverse Events, version 4.0.^18^

### Statistical analysis

Categorical and continuous data values were presented as frequency (percentage) and means. Descriptive statistics were used to summarize demographic, clinical, laboratory, and other related parameters and characteristics of the participants. Association between two or more qualitative variables was examined using Chi-square test. Quantitative data between the two independent groups were analyzed using unpaired t-test. Univariate and multivariate analysis were used to assess the risk that influences calcium levels. Pictorial presentations of the key results were made using appropriate statistical graphs. All P-values presented were two-tailed, and P-values of < 0.05 were considered statistically significant. All statistical analyses were performed using SPSS 24 (SPSS Inc., Chicago, IL).

## Results

### Baseline characteristics

A total of 385 patient records were retrieved from electronic medical records. A total of 114 patients (ZA = 76; DE = 38) were excluded for incomplete laboratory results or alternative use of BTA. Overall, 271 patients were included in this study (ZA = 152; DE = 119), which accounted for a total of 1141 administration encounter (ZA = 776; DE = 365). In both treatment groups, baseline characteristics were similar between patients (Table 1).

**Table 1.** Baseline characteristics.

Characteristics	Denosumab (N = 119)	Zoledronic acid (N = 152)
*Sex*		
Female (%)	76 (63.9%)	76 (50%)
Male (%)	43 (36.1%)	76 (50%)
Mean age (year)	58	55
Mean weight (kg)	73	74
Mean height (cm)	160	162
Baseline creatinine level in µmol/l (mg/dl)	84 (0.95)	67 (0.76)
Baseline corrected Ca level in mmol/l (mg/dl)	2.4 (9.6)	2.34 (9.36)
*Diagnosis*		
Breast cancer	67 (56%)	54 (36%)
Prostate cancer	26 (22%)	20 (13%)
Multiple myeloma	2 (1.7%)	34 (22.4%)
Lung cancer	8 (6.7%)	13 (8.6%)
Renal cell carcinoma	3 (2.5%)	5 (3.3%)
Head and neck cancer	5 (4.2%)	3 (2%)
Colon cancer	2 (1.7%)	2 (1.3%)
Endometrial cancer	1 (0.8%)	1 (0.7%)
Ovarian cancer	1 (0.8%)	1 (0.7%)
Cholangiocarcinoma	1 (0.8%)	1 (0.7%)
Hepatocellular carcinoma	0	2 (1.3%)
Sarcoma	1 (0.8%)	3 (2%)
Pancreatic cancer	0	2 (1.3%)
Mixed (MM/prostate cancer)	0	1 (0.7%)
Others	3 (2.5%)	11 (9.2%)

### Incidence of hypocalcemia and hypercalcemia

**Figure 1.**
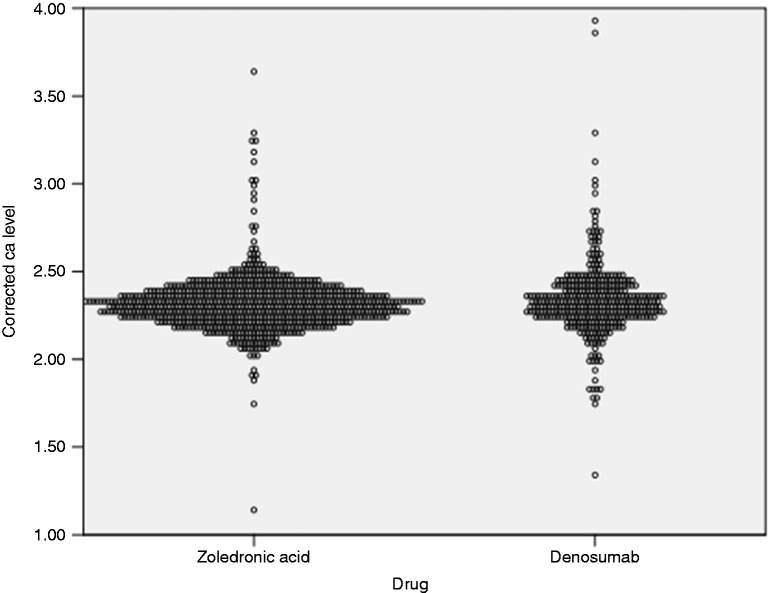
Documented corrected calcium level per each administration visit.

**Figure 2.**
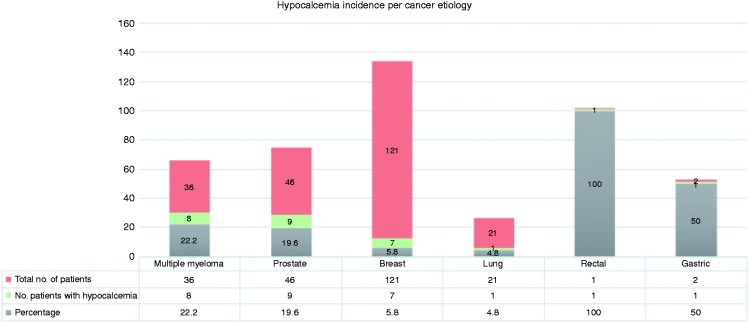
Hypocalcemia incidence per cancer etiologies.

Corrected calcium levels were evaluated on each administration visit. Levels ranged from 1.14 to 3.93 mmol/l (4.56–15.72 mg/dl). The lowest calcium level was reported in ZA group. Figure 1 demonstrates the documented calcium level in each group. Since a patient will receive a bone targeting agent multiple times, the incidence of hypocalcemia was measured per each administration visit. Hypocalcemia incidence was lower among patients receiving ZA compared to those received DE (3.1% vs. 5.5%, OR = 0.55, 95% CI [0.3–1.0]; P = 0.05). It was observed that hypercalcemia was approximately three times more likely to occur in patients receiving DE than ZA (8.5% vs. 3.1% respectively, OR = 2.9, 95% CI [1.68–5.03]; P < 0.0001) (Table 2). Overall, 27 out of 227 patients (11.9%) developed hypocalcemia. Hypocalcemia incident was found among 22% of patients with multiple myeloma, 19% in prostate cancer, 5.8% in breast cancer, 4.8% in lung cancer, 100% in rectal cancer, and 50% in gastric cancer (Figure 2).

**Table 2.** Incidence of hypocalcemia and hypercalcemia among study groups by (patients and administration visits).

	Total no. of patients	No. of administration visits	Hypocalcemia				Hypercalcemia			
			No. of patient with hypocalcemia	% of patients with hypocalcemia	Hypocalcemia incidence per each administration visit	% of hypocalcemia per administration visit	No. of patient with hypercalcemia	% of patients with hypercalcemia	Hypercalcemia incidence per each administration visit	% of hypercalcemia per administration visit
Denosumab	119	365	12	10.10	20	5.50	18	15.20	31	8.50
Zoledronic acid	152	776	17	11.20	24	3.10	13	8.50	24	3.10

### Sub-group analysis

**Figure 3.**
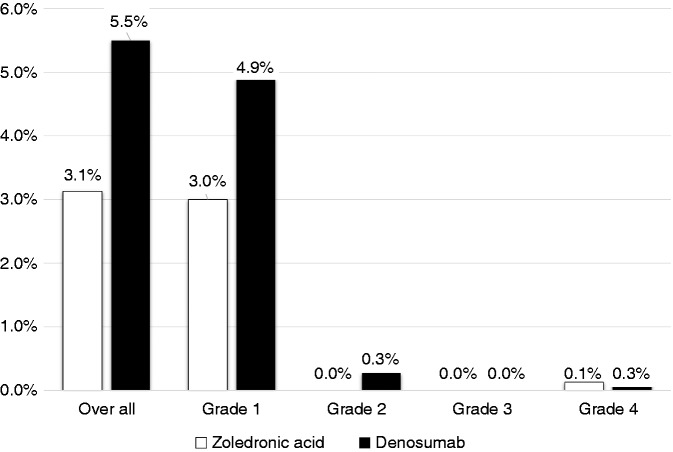
The grade of hypocalcemia events.

**Figure 4.**
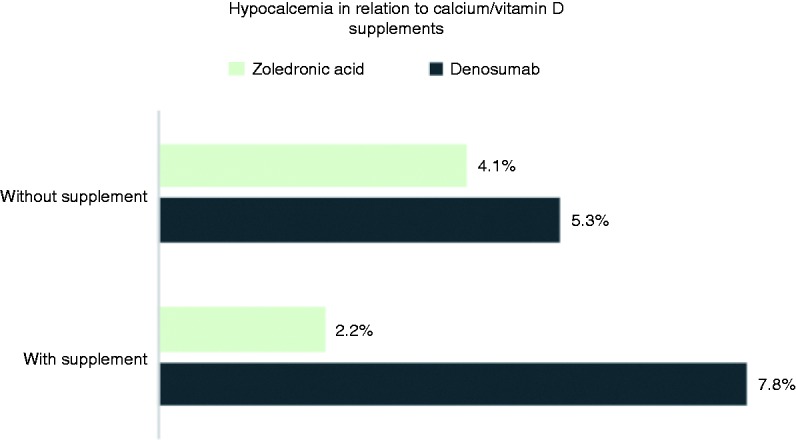
Incidence of hypocalcemia in relation to calcium/vitamin D supplementation.

Most of hypocalcemia events were grade 1 (ZA = 3%, DE = 4.9%) (Figure 3), and no fatal events were reported. It was observed that approximately 60% of patients did not receive calcium/vitamin D supplements. In the ZA group, 46.8% of patients received supplements compared to 26.6% in the DE group (P < 0.0001). In our observation, hypocalcemia incidence was 1.3 times more likely to occur in patients who did not receive supplements compared to those who did (4.5% vs. 3.3% respectively; OR: 1.3, 95% CI [0.73–2.60], P = 0.32) (Figure 4).

## Discussion

In this study, both treatment groups’ baseline characteristics were generally similar between patients. It was observed that a higher number of patients with multiple myeloma received ZA (22.4%) compared to DE (1.7%). This could be explained by the fact that ZE was first approved by FDA in multiple myeloma back in 2002, while DE was approved recently in 2017. Hypocalcemia was seen more frequently in DE group compared with ZA group (5.5% vs. 3.1%). Despite it was statistically insignificant (P-value 0.05), it implies clinical importance toward preventing and monitoring hypocalcemia. Similar findings were reported in a randomized controlled trial in patients with prostate cancer, where more events of hypocalcemia occurred in DE group (13%) than in ZA group (6%).^16^ In another study, reported hypocalcemia events were more common with DE than ZA among patients with multiple myeloma (14% vs. 11%, respectively).^19^ The substantial effect of DE on lowering calcium levels has been justified by the fact that RANKL inhibitors are more potent at reducing the release of calcium into the bloodstream via its new added impact on reducing bone turnover.^20^ We further analyzed the hypocalcemia events based on severity and found that most of them were Grade 1 (3.6%), 4.9% with DE versus 3.0% with ZA. Unlike what was reported by Body et al.,^15^ where Grade 2 hypocalcemia was the dominant. Results from a meta-analysis of seven randomized controlled trials showed a significantly higher risk of all-grade hypocalcemia among DE versus control groups with a relative risk of 1.93.^21^ Higher hypocalcemia events were observed among patients with prostate cancer 9 out of 46 and multiple myeloma 8 out of 36. This finding was also observed by Zuradelli et al.,^13^ who found a correlation between hypocalcemia with breast and prostate cancer (P = 0.006). Despite this finding, we were unable to explain the association between cancer etiology and risk of hypocalcemia, suggesting further studies to consider this aspect.

In our analysis, factors that could influence calcium level were evaluated. It was observed that patients receiving calcium/vitamin D supplements were 16% less likely to develop hypocalcemia (P = 0.39). In a retrospective analysis, the administration of calcium/vitamin D supplements led to a 40% reduction in hypocalcemia incidence among patients who received DE.^15^ In both of our study groups, the incidence of hypocalcemia was 1.1 times higher in those who did not receive supplements. In ZA group, a higher percentage of patients (46.8%) were receiving supplementation compared to patients in DE group (26.6%) which was statistically significant. This finding could be best explained by the higher number of patients on ZA compared to DE. Additionally, since ZA is administered in an inpatient setting unlike DE, there is a better recognition of the need for supplements. Furthermore, the higher percentage of patients in ZA group receiving supplements might justify the raise in hypocalcemia incidence in DE group. The retrospective design did not allow us to assess patient compliance to calcium/vitamin D supplements which made our observations depend solely on supplements dispensing status. In addition, our analysis was based on laboratory calcium levels, yet it was not correlated clinically as an adverse effect due to insufficient reporting/documentation. Moreover, we collected a 12-month worth of data which is not enough to detect the effect of long-term administration of BTAs on calcium levels. Therefore, a prospective study, ideally randomized controlled trial, is granted to assess the long-term effect of BTAs on calcium level and its relationship with disease severity.

## Conclusion

Our findings add to the growing evidence of the impact of BTA, DE, and ZA on calcium levels. A higher incidence of hypocalcemia was observed with DE in comparison to ZA. This result strongly supports the importance of preventative measures when administering BTA. Close monitoring of calcium level and adequate supplementation with calcium and vitamin D should be warranted to reduce the risk of hypocalcemia. Further studies would be useful to assess the long-term effects on calcium level.
